# Predicting Boar Sperm Survival during Liquid Storage Using Vibrational Spectroscopic Techniques

**DOI:** 10.3390/biology13100763

**Published:** 2024-09-26

**Authors:** Serge L. Kameni, Bryan Semon, Li-Dunn Chen, Notsile H. Dlamini, Gombojav O. Ariunbold, Carrie K. Vance-Kouba, Jean M. Feugang

**Affiliations:** 1 Department of Animal and Dairy Sciences, Mississippi State University, Starkville, MS 39759, USA; 2 Department of Physics and Astronomy, Mississippi State University, Starkville, MS 39759, USA; 3 Department of Biochemistry, Molecular Biology, Plant Pathology, and Entomology, Mississippi State University, Starkville, MS 39759, USA

**Keywords:** extended semen, hog, machine learning, near-infrared spectroscopy, Raman spectroscopy, sperm parameters, semen preservation

## Abstract

**Simple Summary:**

Artificial insemination (AI) is a reproductive technique routinely used in livestock to deliver sperm directly to the cervix or uterus to achieve pregnancy. In swine, AI is performed with chill-stored sperm displaying minimum criteria evaluated using traditional procedures; however, variegated fertility appeals for additional tools for sperm evaluation. To enhance sperm evaluation, we assess the potential of near-infrared (NIR) and Raman spectroscopy in addition to traditional techniques to monitor boar sperm quality during 10-day chill-storage. Sperm samples showed differential responses to storage, with sperm quality nearly maintained in some samples, whereas it sharply deteriorated in others. Better than NIR, Raman spectral profiles obtained from freshly extended samples efficiently predict sperm survival to storage, making the technique a promising tool for in-depth assessment of sperm samples.

**Abstract:**

Artificial insemination (AI) plays a critical role in livestock reproduction, with semen quality being essential. In swine, AI primarily uses cool-stored semen adhering to industry standards assessed through routine analysis, yet fertility inconsistencies highlight the need for enhanced semen evaluation. Over 10-day storage at 17 °C, boar semen samples were analyzed for motility, morphology, sperm membrane integrity, apoptosis, and oxidative stress indicators. Additionally, machine learning tools were employed to explore the potential of Raman and near-infrared (NIR) spectroscopy in enhancing semen sample evaluation. Sperm motility and morphology gradually decreased during storage, with distinct groups categorized as “Good” or “Poor” survival semen according to motility on Day 7 of storage. Initially similar on Day 0 of semen collection, “Poor” samples revealed significantly lower total motility (21.69 ± 4.64% vs. 80.19 ± 1.42%), progressive motility (4.74 ± 1.71% vs. 39.73 ± 2.57%), and normal morphology (66.43 ± 2.60% vs. 87.91 ± 1.92%) than their “Good” counterparts by Day 7, using a computer-assisted sperm analyzer. Furthermore, “Poor” samples had higher levels of apoptotic cells, membrane damage, and intracellular reactive oxygen species on Day 0. Conversely, “Good” samples maintained higher total antioxidant capacity. Raman spectroscopy outperformed NIR, providing distinctive spectral profiles aligned with semen biochemical changes and enabling the prediction of semen survival during storage. Overall, the spectral profiles coupled with machine learning tools might assist in enhancing semen evaluation and prognosis.

## 1. Introduction

Whether in vivo or in vitro, successful reproduction mainly relies on high-quality gametes. In routine practices, selecting fresh semen samples for processing represents a critical step as, alongside other factors (i.e., stress, disease, and environmental factors), it determines the final quality of preserved semen samples and, ultimately, fertility outcomes following artificial insemination (AI) [1]. In commercial boar studs, AI is performed with extended (or liquid) semen samples of healthy sires satisfying motility (≥70%) and morphological (≥80%) thresholds [2]. However, growing evidence supports that motile sperm cells with standard shape might not be fully functional and feature some discrepancies, such as abnormal chromatin condensation and DNA damage [3,4], and fertilization by sperm with abnormal chromatin condensation may lead to early embryonic loss or the development of genetic diseases with subsequent loss in profitability. Therefore, thorough assessments of semen samples are necessary to ensure optimal fertility and prevent economic losses associated with impaired fertility (lower conception rate and litter size) [2].

Improving data-driven ejaculate selection combines simple techniques (e.g., hemocytometer and photometer/spectrophotometer) with more sophisticated ones (e.g., flow and computerized cytometry). Unlike simple (and subjective) techniques, sophisticated techniques provide more rapid, reliable, and objective assessments (e.g., concentration, motility, viability, and abnormalities). Unfortunately, sophisticated instruments present important limitations. For examples, flow cytometry which combines a myriad of labeled procedures to evaluate sperm parameters could be expensive and time-consuming, requiring highly trained personnel for sample analyses [5,6,7,8]. In addition, though more affordable and user-friendly, providing more practical and widely on-field applications to further optimize male selection and improve AI results through rapid and objective assessment of sperm quality [9,10], the computer-assisted sperm analyzer (CASA) suffers from the lack of standardization and variable approaches in its development [11]. Though these techniques have contributed to the advancement of assisted reproductive technologies, especially in semen analysis, they do not seize the whole picture of the frame, especially the biochemical subtleties that vibrational spectroscopy might unravel [12,13,14]. Semen quality assessment represents a core step for successful AI and CASA parameters are the gold standard in the selection of semen samples for storage and/or subsequent insemination. This process. however, still results in AI doses with variable and sometimes suboptimal fertility outcomes (e.g., small litter size) [9], suggesting routine semen quality estimates are not sufficiently sensitive to characterize semen samples. Therefore, there is still a desperate need for fast, easy-to-implement, and non-destructive methods for decisive sample evaluation at multiple stages of semen handling, which is essential for improving male reproductive performance.

Recently, there has been a growing interest in using spectroscopy to assess the characteristics of biological samples [15,16]. The development of optics, miniaturization, and information technology/chemometrics have made techniques such as Raman and near infrared (NIR) spectroscopy valuable tools, capable of providing molecular details of a sample in a short time frame with a spatial resolution in the sub-micrometer range [17,18]. Raman and NIR spectroscopy are vibrational, versatile, non-invasive, and non-destructive techniques, requiring less to no sample preparation that yield detailed information about chemical structure [19]. Both techniques generate unique spectral footprints that can be used to identify vibrating molecules, and their specificities and possible combinations may be useful for semen analysis. Raman spectra generated by light scattering of chemical bonds within the sample generally have sharper and better-resolved peaks than NIR spectra, allowing qualitative and quantitative analyses of little-known to unknown samples [20,21,22]. Indeed, Raman measurements targeting non-polar molecular bonds (e.g., C-C) are gathered within the width of the monochromatic laser light used for sample excitation. In contrast, the NIR measurements are broader and target polar molecular bonds [18,23]. Hence, both techniques have been used in situ, in vivo, or in vitro across organic and inorganic samples [15,24,25]; however, little is known about their use to assess sperm functionality.

Few studies have been conducted to decipher spermatozoa quality using Raman or infrared spectroscopy. Raman spectroscopy has shown effectiveness at sorting bovine X- and Y-chromosome-bearing spermatozoa with an accuracy like that achieved with fluorescence-based cell sorting [22,26,27]. Other studies demonstrated the efficacy of micro-Raman spectroscopy to capture non-labeled sperm macromolecules (DNA, proteins, and carbohydrates), potentially enabling discrimination of spermatozoa bearing damaged and uncompacted DNA [12,28,29,30]. A specific Raman spectrum band has been assigned as an indicator of DNA damage in sperm, regardless of the insult source [17,21,31,32,33]. Concurrently, peaks revealed by the analysis of infrared spectra have been correlated to sperm concentration and kinematics [24]. Other studies showed that spectral signatures from the DNA backbone and endogenous proteins vary in response to induced oxidative damage and chromatin decondensation, suggesting that spectral fingerprints are potent indicators for sperm evaluation quality [21,34]. These promising results support using vibrational spectroscopy as an adjunct for more efficient semen analysis. However, the benefits of vibrational spectroscopy have not been fully explored in previous studies since the approach was generally invasive, with a limited number of spermatozoa.

To our knowledge, none of the spectroscopic studies focused on suspended live spermatozoa to provide a rapid, non-invasive, and high-throughput technological approach for semen analysis and diagnosis that would immediately benefit users in farms and reproductive medicine clinics. Here, we used Raman and NIR spectroscopy as complementary tools for routine real-time measurement of extended boar semen to generate spectral profiles based on semen quality during prolonged cooled storage.

## 2. Materials and Methods

All chemicals and reagents were purchased from Sigma Aldrich (St. Louis, MO, USA), except where otherwise indicated.

### 2.1. Semen Samples

AI-doses (*n* = 29) of freshly extended semen of proven fertile Duroc boars (1–2.5 years old) were obtained from a commercial boar stud (Prestage Farms, West-Point, MS, USA). Each dose was an equal pool of spermatozoa from two boar ejaculates (*n* = 58, one ejaculate per boar), extended in the NUTRIXcell+ (IMV Biotechnologies, Brooklyn Park, MN, USA). All semen doses were transported within 30 min to the laboratory for experiments. Semen doses were aliquoted into plastic tubes filled to the top, tightly closed, and stored (16–18 °C) for up to 10 days after collection (= day 0). Each tube of semen aliquot (one per replicate or dose) was subjected to daily motility analysis, Raman spectroscopy, and Near-infrared spectroscopy analyses, starting from day 0. Two aliquots (100 × 10^6^ cells each) of each semen dose were centrifuged (900× *g*; 5 min, 20–22 °C) to pellet the sperm cells, which were washed twice with PBS and saved at −80 °C for total antioxidant and lipid peroxidation assays.

### 2.2. Sperm Motility Analysis

On experiment day, semen samples stored at 16−18 °C were incubated for 15 min at 37 °C before motility and morphology assessment (sperm quality), as previously described [13,35]. Sperm motility was evaluated using a CASA system (CEROS II, IMV Biotechnologies) operating at 60 Hz (60 frames/s), with settings of head size from 10 to 50 μm, and minimum tail brightness of 86. Motile sperm were set for average path velocity (VAP) > 45 μm/s and a straight-line velocity (VSL) > 5 μm/s. Sperm progressivity had a VAP > 45 μm/s and straightness > 45%. The proportions of total motile (TM), progressive motile (PM), normal morphology (Nmorph) spermatozoa, and various abnormalities parameters (bent tails, coiled tails, distal and proximal droplets) were recorded for analyses. Kinematic parameters (curvilinear velocity—VSL, straight-line velocity—VSL, and average path velocity—VAP) were also analyzed. Each experiment consisted of at least four biological samples per day with seven independent replicates (*n* = 29). Additionally, samples that deviated (SD: standard deviation) from the average total motility on Day 7 were classified as “Good” (average + ¾ SD) and “Poor/Bad” (average—¾ SD) cooling surviving semen doses.

### 2.3. Raman Spectroscopy Analysis

Daily, each cool stored semen sample was aliquoted into two tubes (1.5-mL Eppendorf tubes). One tube was kept as is (Extended sample), while the second tube was centrifugated (900× *g*; 5 min, 20–22 °C). The supernatant was transferred to a clean tube (Plasma sample). The corresponding sperm pellet was resuspended in phosphate-buffered saline solution (PBS) for a second wash, followed by removing the supernatant and resuspending the pellet in PBS (Pellet sample). The three sample types were maintained in 1.5 mL Eppendorf tubes filled to the top for immediate Raman analyses following a gentle mixture by agitation. For each sample, the incident light from the Raman probe was meticulously handled and brought to the liquid surface in uncapped sample tubes to collect the Raman spectra. The probe’s distance regulator was used throughout the analyses to ensure consistent space between the sample and the lens. The Raman spectroscopy evaluation was performed with a BWTEK MiniRam BTR-111-785 (B&W Tek Inc., Plainsboro Township, NJ, USA) spectrophotometer device (λ = 785 nm, scan range of 200–1000 nm with a 1.2 nm–10 cm^−1^ spectral–optical resolution, and a wide dynamic range for both absorbance and reflectance spectra). Samples were analyzed in the Raman shift range of +175 to 3100 cm^−1^, with a 785 nm laser power set at 30 mW (10% from 300 mW available) to give an irradiance of about 382 W/cm^2^. The laser spot and irradiance levels were kept constant during Raman spectra acquisition. Signal acquisition was performed at room temperature with a TE-cooled linear CCD array detector with 20 s integration time and in system background (water and PBS) subtraction of all measurements. Raman spectra were collected in triplicate from each sample type. At least four biological replicates were analyzed daily for 11 days (including Day 0). The experiment was repeated on seven independent occasions, generating 957 spectra/sample type.

### 2.4. Near-Infrared Spectroscopy (NIRS) Analysis

Sample types (Extended, Spermatozoa, and Plasma) of each semen replicate were prepared as specified above for Raman spectroscopy and stored at -80ºC until use. Hence, NIRS analysis was conducted on frozen–thawed “Good” and “Poor” samples (n = 5 each), as previously described [36]. Briefly, NIR spectra (*n* = 6 scans/sample type) were collected from 300 µL of each “Good” and “Poor” sample transferred in 1 mm quartz cuvette mounted in an ASD-fiber optic cuvette adapter, using an ASD FieldSpec^®^ 4 IndicoPro^®^ portable spectrophotometer (Malvern Panalytical, ASD Analytical Spectral Devices Inc., Boulder, CO, USA; λ = 350−2500 nm, 50 scans with a 34 ms integration time, approximately 1.4–2.0 nm resolution). An empty cuvette was used to determine the baseline between measurements, and sterile distilled water was used as a reference solution/to monitor dark signal laser drift in the system. Spectral data of each sample type (*n* = 3) were collected on Day 0 and Day 7 and used to evaluate the model performance from a global NIRS dataset of 360 spectra, corresponding to 60 spectra per sample type per day (30 each for the Good and Poor sample types).

### 2.5. Flow Cytometry Analyses

The apoptotic-like changes, viability, and ROS intracellular content of freshly extended (Day 0) and 7-day cooled stored semen samples were evaluated using flow cytometry (Novocyte; Agilent, Santa Clara, CA, USA), with 10,000 events per analysis. Samples were washed twice and diluted with PBS solution to a final concentration of 5 × 10^6^ sperm/mL in a final volume of 0.5 mL. Unstained and single-stained samples for each fluorochrome were used for setting the electronic volume gain. Sperm cells were isolated from total events based on forward and side scatter profiles. Apoptotic-like changes were evaluated using the PE Annexin V Apoptosis Detection Kit according to the manufacturer (BD Pharmingen^TM^, BD Biosciences, Franklin Lakes, NJ, USA). The assessment of sperm viability was performed by evaluating sperm membrane stability through staining with propidium iodide (12 µM PI; Invitrogen, Carlsbad, CA, USA) [37] and intracellular ROS were evaluated through staining with highly sensitive dichlorodihydrofluorescein diacetate (DCFH-DA; Dojindo Molecular Tech, Rockville, MA, USA). Experiments were repeated four times.

### 2.6. Total Antioxidant and Lipid Peroxidation

Frozen sperm pellets, collected on Day 0 and Day 7 of storage from samples classified as Good or Poor, were thawed on ice. Cell lysates were obtained using the complete RIPA buffer (RIPA buffer, sodium orthovanadate, protein inhibitor, Phenylmethanesulfonyl fluoride; 97:1:1:1 *v*/*v*) for total antioxidants and a 5-cycle freeze-thawing (1 min in liquid nitrogen and 5 min in water bath at 37 °C) for lipid peroxidation. Lysates were subjected to total antioxidant capacity (TOAC) or malondialdehyde (MDA) content analyses using the appropriate kits (Antioxidant and TBRAS), as recommended by the manufacturer (Cayman Chemical; Ann Arbor, MI, USA). Experiments were repeated four times.

### 2.7. Data Processing and Statistical Analyses

Sperm characteristic data were tested for normal distribution, followed by appropriate tests to evaluate the storage effect (repeated measures ANOVA and Bonferroni test for pairwise comparisons) and differences between “Good” and “Poor” sample groups (Student Fisher’s *t*-test). A *p*-value less than 0.05 was set as the threshold of significance. All Raman (197–3290 nm) and NIR (350–2500 nm) raw data were preprocessed for high-frequency noise removal (only Raman spectral data; boxcar averaging), baseline variation removal (standard normal variate), linear detrending (polynomial order 1), first derivative (gap size-25/Segment size-19), and Savitzky–Golay smoothing (polynomial order-1/symmetric kernel points-12, using the “prospectr” package) [38]. Chemometric analyses were conducted using the “mlr” package of the R suite [39] to simultaneously train and test three conventional machine learning models viz. support vector machine (SVM), linear discriminant analysis (LDA), and partial least squares (PLS). The Raman spectral data were combined due to the small sample size, while the NIR spectral data were grouped according to sample types and storage duration (Day 0 and Day 7). The “Boruta” package and leave-one-out cross-validation were applied for variable selection and model calibration, respectively [40], and predictive metrics of the models were computed. Principal component analysis (PCA) was performed on both Raman and NIRS spectral data as previously described [36,41] using Unscrambler^®^ X v.10.5 program (CAMO Analytics, Osla, Norway).

## 3. Results

### 3.1. Sperm Motility: Differential Response of Semen Doses to Storage

**Figure 1:**
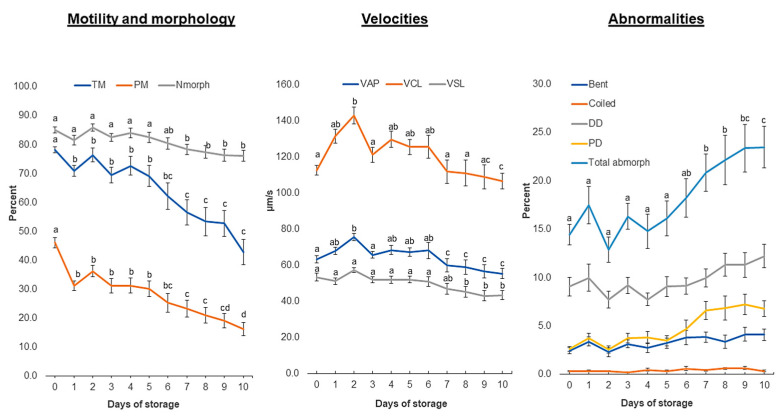
Evolution of boar sperm parameters during prolonged cooled storage. Extended boar semen samples were stored at 17 °C for up to 10 days. TM: total motility; PM: progressive motility; Nmorph: normal morphology; VAP: average path velocity; VCL: curvilinear velocity; VSL: straight-line velocity; Abmorph: abnormal morphology. Different letters (a, b, c, d) denote significant difference between days of storage, *p* < 0.05.

The motility, kinematics, and morphology data patterns during cooled storage are summarized in Figure 1. The proportions of total motility, progressive, and morphologically normal spermatozoa gradually and significantly (*p* < 0.05) decreased from 78.1 ± 1.0%, 46.13 ±1.7%, and 85.0 ± 1.0%, respectively, on the collection day (Day 0) to 42.8 ± 4.3%, 16.2 ± 2.3%, and 76.0 ± 1.9%, on Day 10 of storage. Similar profiles (*p* < 0.05) were observed with other kinematic parameters (VSL, VSL, and VAP). In contrast, the proportion of combined sperm abnormalities (bent tails, coiled tails, distal and proximal droplets) was significantly increased (*p* < 0.05), with the distal droplet being the major contributor.

**Figure 2:**
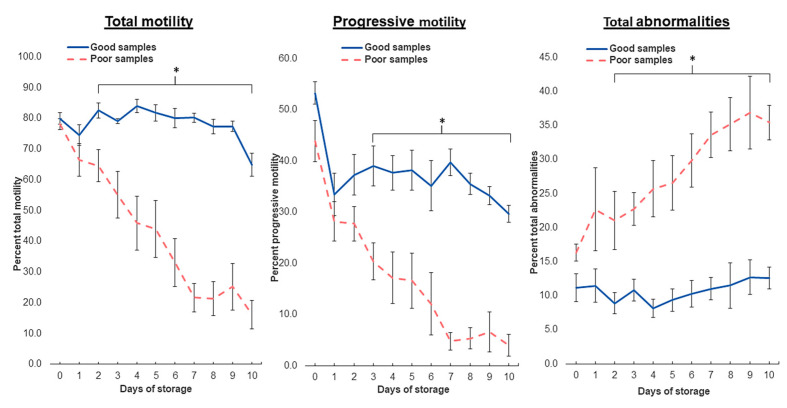
Evolution of boar sperm parameters of Good and Poor semen doses during cooled storage. Extended boar semen samples were stored at 17 °C for up to 10 days. * denote significant differences between Good and Poor within the same day of storage, *p* < 0.05.

The present study showed significant variability among samples during cooled storage, with individual semen samples showing highly dissimilar amplitudes in the decreased trends, particularly for total motility, in which a variability peak was observed on Day 7. Therefore, we identified, based on average TM on Day 7, two distinct groups of semen samples that were considered “Good” (TM > mean + ¾SD) and “Poor” (TM < mean − ¾SD). The average data of Good and Poor semen are shown in Figure 2. The two groups of samples revealed significantly different (*p* < 0.05) data on sperm motilities and total abnormalities from Day 3 of storage. The highest significant differences between the Good and Poor semen samples were found on Day 7 for total motility (80.2 ± 1.4% vs. 21.69 ± 4.6%), progression (39.7 ± 2.6% vs. 4.7 ± 1.7%), and total abnormalities (11.0 ± 1.6% vs. 33.6 ± 3.4%). Moreover, the mean values of VAP, VCL, and VSL in the Poor samples were significantly lower (*p* < 0.05) than those in the Good Survival samples (40.4 ± 7.5 µm/s vs. 74.9 ± 2.6 µm/s, 81.9 ± 15.4 µm/s vs. 135.2 ± 6.0 µm/s, and 31.3 ± 5.8 µm/s vs. 59.0 ± 1.8 µm/s, respectively). However, these velocity parameters in the Good Survival samples were comparable between Day 0 and Day 7 (*p* > 0.05).

### 3.2. Vibrational Spectroscopy Fingerprints and Classification of Samples

The preprocessing of spectral datasets with the “Boruta” algorithm led to the selection of 79 and 60 variables for Raman and NIR spectroscopy, respectively. Table 1 shows the statistical summary of Raman and NIR spectroscopy prediction models. The LDA appeared as the suitable model with the highest accuracy, sensitivity, and specificity percentages (72–83%) to analyze Raman spectral data in Extended and Sperm sample types. Meanwhile, on average, the LDA and SVM algorithms were the best-performing models for most NIRS datasets. Interestingly, the best discrimination results, regardless of the spectroscopic tool and the algorithms tested (SVM, LDA, and PLS), were obtained with the Plasma sample type, showing the highest accuracy, sensitivity, and specificity.

**Figure 3:**
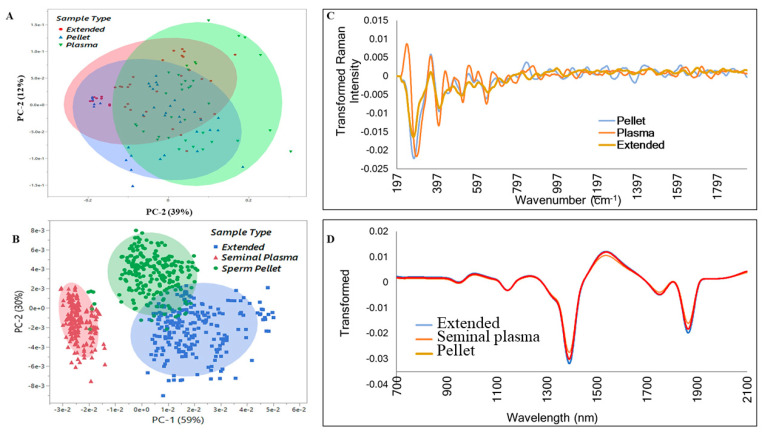
Principal component analysis (PCA) score plots and transformed spectra of different sample types: (**A**) Raman PCA score plot. (**C**) Transformed Raman spectra. (**B**) Near-infrared PCA score plot. (**D**) Transformed near-infrared spectra.

**Figure 4:**
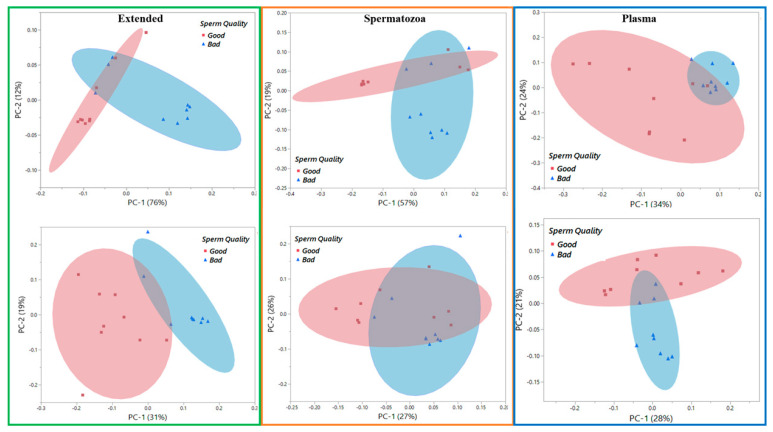
Principal component analysis score plots of Raman data of different sample types from boar semen. Raman spectra were collected on (**upper** panel) Day 0 and (**bottom** panel) Day 7 from Good and Bad/Poor semen doses.

**Figure 5:**
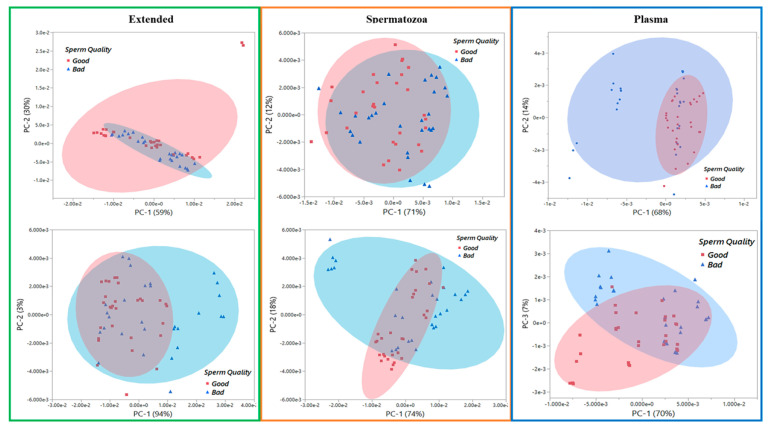
Principal component analysis score plots of near-infrared data of different sample types from boar semen. Near-infrared spectra were collected on (**upper** panel) Day 0 and (**bottom** panel) Day 7 from Good and Bad/Poor semen doses.

**Figure 6:**
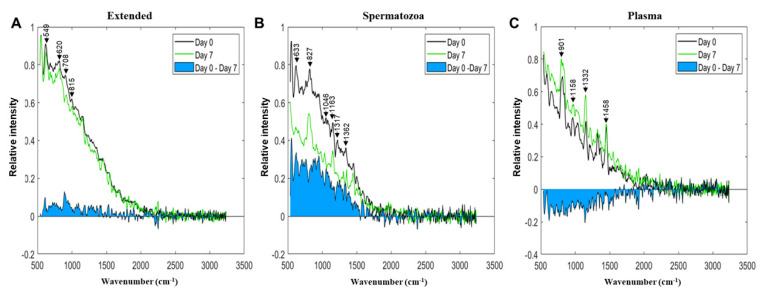
Average Raman spectra collected on Day 0 and Day 7 of boar semen storage: spectra from (**A**) Extended, (**B**) Spermatozoa, and (**C**) Plasma sample types were filtered out using the Savitzky–Golay filter (order 3 and frame length 23) and normalized to the highest spectral value from each sample group type.

Figure 3 represents the principal component analysis (PCA) score plots of the transformed raw data, enabling the visualization of trends and outliers of the spectral datasets. The NIR spectroscopy was better suited for sample-type clustering (Figure 3). Interestingly, Raman was better fitted for sample grouping i.e., Good vs. Poor (Figure 4) compared to NIR (Figure 5). The PCA score plots show the ability of the Raman spectroscopy to distinguish semen samples based on sperm quality in the Extended, Spermatozoa, and Plasma sample types on Day 0. Consequently, further analyses were performed only with Raman spectral datasets having the highest classification performance, irrespective of the sample type. The average Raman spectra (filtered and normalized) of Day 0 and Day 7 of all semen doses evaluated in this study are summarized in Figure 6, showing Extended semen spectra with the corresponding Spermatozoa and Plasma ones. These spectral profiles, with various prominent peaks summarized in Table 2, provide an array of Raman-active compounds found in different sample types during storage. Figure 6 also shows that the Extended and Sperm sample types had higher Raman intensities on Day 0 than on Day 7, with the most notable intensity differences seen with the Sperm sample type. The inverse figure was noticed with the Plasma sample type, the spectra profile of Day 7 showing higher intensity compared to that of Day 0.

**Figure 7:**
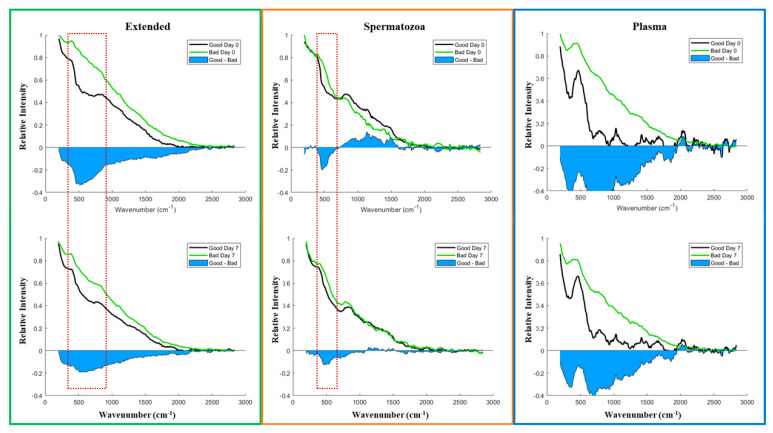
Average Raman spectra of different sample types from Good vs. Bad/Poor boar semen doses. Raman spectra were collected on (**upper** panel) Day 0 and (**bottom** panel) Day 7, filtered out using the Savitzky–Golay filter (order 3 and frame length 23), and normalized to the highest spectral value from each sample group type.

**Figure 8:**
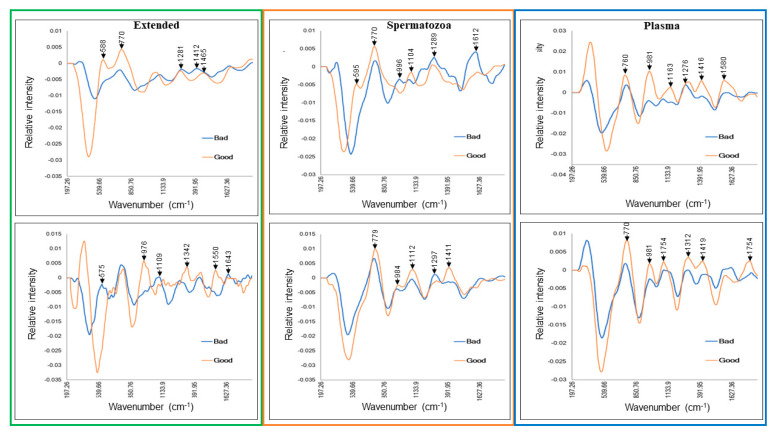
Transformed Raman spectra of different sample types from Good vs. Bad/Poor boar semen doses. Raman spectra were collected on (**upper** panel) Day 0 and (**bottom** panel) Day 7.

Most importantly, Raman spectroscopy could distinguish biochemical heterogeneities between Good and Poor semen doses. Both groups displayed distinct spectra on Day 0 and Day 7 of storage (Figure 7). Comparisons of both groups to detect likely pre-existing compounds on Day 0 revealed interesting Raman shift changes in the Extended (266–866 cm^−1^), Spermatozoa (333–666 cm^−1^), and Plasma (250–2500 cm^−1^) sample types (Figure 7). The same figure was observed on Day 7. The transformed spectra in Figure 8 accentuate the spectral response to chemical changes in samples on Day 0 and Day 7 and reveal several sample-type- and storage-time-dependent peaks (Table 3).

### 3.3. Sperm Quality Attributes

The sperm characteristics of the Good and Poor survival samples were assessed on Day 0 and Day 7 and the results are summarized in Table 4. On Day 0, the percentages of early apoptotic cells, damaged membrane cells, and cells with intracellular accumulation of ROS were significantly higher in the Poor samples than in the Good ones (*p* < 0.05). On Day 7, Poor Survival semen showed a significantly (*p* < 0.05) higher proportion of membrane-damaged cells and higher fluorescence intensities than Good Survival with all parameters. Other parameters were comparable on Day 7 (*p* > 0.05). For each parameter analyzed with flow cytometry, Good Survival samples showed no difference between Day 0 and Day 7; whereas there were significant differences for Poor samples. Further, there was no significant difference in MDA content between Good and Poor samples on Day 0; however, on Day 7, the Poor Survival samples showed a higher (*p* < 0.05) MDA concentration than the Good ones. In contrast, the Good Survival doses displayed higher (*p* < 0.05) TAOC on Day 0 but were comparable to their Poor counterparts on Day 7. Also, the TAOC:MDA ratio revealed a significant difference in favor of Good Survival semen, irrespective of the storage time (*p* < 0.05).

## 4. Discussion

Despite the improvement of semen selection criteria for storage and subsequent breeding, the gradual decline of sperm quality during storage constitutes a major drawback for the optimal implementation of assisted reproductive technologies, especially AI. Furthermore, there is a sire-to-sire ejaculate variation in withstanding storage [42,43] with a subsequent impact on fertility, hence the need for alternative tools to unveil features not apprehended by routine procedures. Therefore, the present study used conventional analysis (CASA) associated with vibrational spectroscopy to monitor sperm quality during 10-day storage.

In the present study, sperm parameters assessed by CASA deteriorated during storage. Such a report aligns with the well-documented knowledge of the progressive decline of sperm attributes during prolonged storage [44,45,46], correlating with reduced farrowing rates and litter size [47,48]. In pigs, the sperm-rich faction of the raw semen is diluted with commercial or homemade extenders for cooled preservation, increasing the number of semen doses per single ejaculate and the lifespan of spermatozoa. Despite the lower temperature imposed (16–18 °C) to reduce the metabolism of spermatozoa during storage, they are still subject to attacks linked to aging, insufficient protective factors of the seminal plasma, and increased exposure to light, which generally reduce the proportion of viable sperm [47]. Many studies have reported the harmful effects of the accumulation of reactive oxygen species (ROS) in boar spermatozoa during cooled storage, altering proteins involved in sperm metabolism [49] and reducing sperm motility and morphology [50].

The present study showed significant variability among samples during cooled storage, allowing us to distinguish two groups of semen doses: Good and Poor. Notably, both groups of semen met the industry standards for AI at collection, meaning they were equally prepared for breeding. A similar observation was obtained with boar semen extended in Beltsville Thawing Solution during 120 h of storage at 17 ± 1 °C [51]. Therefore, we speculated that the heterogeneous dynamics of semen doses during cooled storage could explain the observed discrepancy in field fertility. This speculation was supported by a recent study demonstrating that breeding with liquid-stored semen containing high-motility spermatozoa significantly increases litter size [51]. It is generally admitted that breeding with short-preserved diluted boar semen leads to better fertility outcomes than that with semen stored for a long time. In the present study, all semen doses were diluted with the same commercial extender (NUTRIXcell+), eliminating the possibility of the extender differently affecting semen (sperm and seminal plasma) doses during storage. Therefore, we speculated that Good and Poor semen might reflect semen features (i.e., membrane fluidity, cholesterol/phospholipid ratio and intracellular components, ionic balance, and metabolism) that are exacerbated during storage by interactions with various semen components and environmental stressors (e.g., light). Nonetheless, the specific implications of extender dilution and interactions with semen remain understood.

Raman and near-infrared (NIR) spectroscopy are two versatile tools that have found numerous practical applications in various fields of (bio)sciences [15,18,52], due to their high specificity, high-throughput analysis, non-destructive nature, and potential for on-field applications [19]. These attributes make both tools attractive for rapid confirmatory identification of various biofluids, including semen [15,19,53]. However, applying both Raman and NIR spectroscopy for semen diagnostics requires further method development and validation for realistic applications in reproductive technology. Because light and matter interact in many ways, we thought both Raman and NIR spectroscopy would measure this interaction to produce spectra from liquid semen samples and permit detailed investigation of semen doses.

Here, we first tested whether the prediction models based on the liquid semen would be reliable for the desired application. A total of 79 and 60 spectral variables were selected for Raman and NIR datasets, respectively, with Boruta package R, and subjected to leave-one-out cross-validation before prediction modeling. This approach has been shown to be very effective in analyzing small-size samples, reaching strong classification performance [36,41]. The prediction models respond differently according to spectral data and sample types, with LDA being most suitable to analyze Raman spectral data in Extended and Sperm sample types, while LDA and SVM algorithms were more appropriate for NIRS datasets. SVMs, LDA, and PLS are three algorithms successfully applied in various biological samples for sample classification [15,36,54]. The different rationales behind the development and implementation of these algorithms might explain their variable ability to discriminate samples. Regardless of the spectroscopic tool and the algorithms tested (SVM, LDA, and PLS), the Plasma sample type emerged to be more informative, showing the highest prediction parameters (accuracy, sensitivity, and specificity). A previous study using raw sperm showed that seminal plasma was the main contributor to Raman spectra [55]. It is worth mentioning that semen doses analyzed in the current study were processed without removal of the seminal plasma, suggesting that, even with the dilution with an extender, Raman spectroscopy is sufficiently sensitive to capture the divergent dynamics of associated molecules during storage. Indeed, numerous studies have reported seminal plasma as a reservoir of potential biomarkers (e.g., proteins, metabolites, miRNAs) of semen quality [35,56,57,58].

The NIR spectroscopy was better suited for sample type clustering, while Raman spectroscopy evidenced a superior ability to discriminate sample according to sperm quality (Good vs. Poor), emphasizing the potential use of Raman spectroscopy as a promising alternative technique for semen analysis, provided that an effective prediction model for sample classification is established. Of interest, the usage of Raman spectroscopy allowed us to confirm semen samples classification as Good or Poor semen quality from Day 0, which could enable rapid diagnosis of semen doses and contribute to their optimal management in breeding operations. For all sample types, the average Raman profile showed different intensities between the spectra of Day 0 and Day 7, with several peaks. These differences mainly were observed within the 600–1500 cm^−1^ range, corresponding to the vibration modes of proteins, nucleic acids, amino acids, carbohydrates, and lipids. This range belongs to the so-called fingerprint region of the entire Raman spectrum (400–1800 cm^−1^), where most of the hydrogen-bonded (i.e., C=O, C-C, and C-N) protein side groups and the protein backbone show absorbance bands [59,60]. Several peaks detected in this range have been associated with various regions of the sperm head (e.g., ~1094, ~1180, and ~1420 cm^−1^), mid-piece (e.g., ~743, ~1109, and ~1364 cm^−1^), and tail (e.g., ~1003, ~1288, and ~1607 cm^−1^) [61]. Focusing on the spectra of the Plasma sample type which exhibited negative peak differences, we hypothesized that various biochemical components of sperm were released into the medium during storage, leading to opposite spectral displays between Day 0 and Day 7.

Raman spectroscopy showed potential to discriminate between Good and Poor semen doses from Day 0 with spectral shift changes in Extended (range of 266 cm^−1^ to 866 cm^−1^) and Spermatozoa (333 cm^−1^ and 666 cm^−1^) sample types mainly corresponding to the vibration modes of nucleic and amino acids. As for the Plasma type, the difference between spectra of Good and Poor samples was spread on a broader wavenumber range (250–2000 cm^−1^). This region corresponds to the spectral range of Raman detection of the major categories of biomolecules (proteins, carbohydrates, lipids, and derivatives), hence highlighting the abundance of Raman-sensitive molecules in the Plasma sample type. The transformed spectra revealed a similar pattern with all detected peaks, associated with known assigned wavenumbers of spermatozoa [19], being sample-type- and storage-time-dependent. It is therefore possible to envision that time-dependent identification of wavenumbers specific to each sample type may contribute to the development of biomarker candidates to enable the selection of robust and more resilient extended semen for well-planned cooled storage. Indeed, previous studies have shown the potential of Raman spectroscopy to detect damage to sperm DNA and mitochondria. For example, wavenumbers ~1420 and ~1612 cm^−1^ have been associated with sperm head damage following UV exposure [19] and semen manipulation [28], respectively, while wavenumbers 1050 and 1095 cm^−1^ were proposed as markers for monitoring oxidative damage in human sperm DNA [21]. In another study, wavenumbers ~ 751, ~788, and ~2850–3000 cm^−1^ helped to assess the mitochondrial status of sperm [30].

The percentages of early apoptotic cells, membrane-damaged cells, and cells with intracellular accumulation of ROS were significantly higher in the Poor samples compared to Good ones on Day 0, indicating that despite both sample groups meeting the thresholds (motility ≥ 70% and morphology ≥ 85%) for storage, other intrinsic sperm characteristics participate in the ability of semen to withstand storage and may condition the sperm motility dynamics. Furthermore, proteomic and metabolomic analyses of boar spermatozoa with different preservation abilities (grouped according to motility) have revealed differently abundant proteins and metabolites specific to each group [62,63]. Being Raman-active, many of these proteins and metabolites might have accounted for the Raman classification of the Good and Poor samples portrayed in the present study.

Furthermore, Poor Survival samples demonstrated a higher MDA content on Day 7, while Good Survival displayed greater TOAC on Day 0, suggesting that Poor Survival samples are more prone to lipid peroxidation during storage while the TAOC content of Good Survival samples may confer them a superior ability to withstand storage. This was in accordance with the greater ratio TAOC:MDA of Good Survival samples compared to Poor ones on Days 0 and 7, indicating different levels of oxidative stress experienced by both semen groups. Previous studies reported that post-storage sperm quality correlates with intracellular oxidants and antioxidants [50,64]. With the MDA and antioxidant molecules containing vibrating bonds and thus being Raman sensitive, it is more likely that the dynamics of lipid peroxidation and TOAC might elucidate the ability of Raman spectroscopy to discriminate between Good and Poor Survival semen. In addition, the significantly different ratio of TAOC:MDA between Good and Poor Survival semen samples at each time point might account for this discrimination.

At this stage of the work, it is essential to mention that the present study differs from previous ones, applying Raman spectroscopy on fixed or dried spermatozoa. These experimental approaches can cause variations in light scattering, compromising comparisons of detected peaks of vibrational compounds, as previously reported [65]. Despite these potential pitfalls and experimental artefacts, the present study provides a fingerprint of extended boar semen while depicting differential phenotypic spectral profiles to enable rapid screening for prolonged cooled storage.

## 5. Conclusions

The present study is likely the first applying vibrational Raman and NIR spectroscopy for the real-time monitoring of liquid semen. The results indicate that fertile boar semen, classified as having Good and Poor storage survival, exhibits unique spectral fingerprints upon collection and after seven days of storage. These spectral fingerprints coinciding with differentially detected sperm defects in the two semen groups provide new clues to reliably identify Good or Poor Survival semen from the day of collection. The findings open new horizons for optimal semen management for consistently high fertility outcomes—Poor Survival semen samples being used before the Good Survival ones. However, additional research (e.g., fertility related to Poor and Good Survival semen) is needed before implementing this technological approach in the field with any mammalian species.

## Figures and Tables

**Table 1:** Performances of predictive models using Raman and Near-infrared spectra from boar semen doses.

Sample and Size	Models	Extended			Spermatozoa			Plasma		
		Accuracy	Sensitivity	Specificity	Accuracy	Sensitivity	Specificity	Accuracy	Sensitivity	Specificity
Raman * (*n* = 36)	SVM	72.2%	61.1%	83.3%	75.0%	72.2%	77.8%	97.2%	100.0%	94.4%
	LDA	75.0%	72.2%	77.8%	77.8%	72.2%	83.3%	91.7%	94.4%	88.9%
	PLS	69.4%	66.7%	72.2%	77.8%	77.8%	77.8%	86.1%	94.4%	77.8%
NIR Day 0 (*n* = 30)	SVM	63.3%	80.0%	46.7%	60.0%	50.0%	70.0%	80.0%	86.7%	73.3%
	LDA	63.3%	56.7%	63.6%	83.3%	66.7%	100.0%	81.7%	70.0%	93.3%
	PLS	55.0%	66.7%	43.3%	73.3%	56.7%	90.0%	55.0%	40.0%	70.0%
NIR Day 7 (*n* = 30)	SVM	88.3%	86.7%	90.0%	61.7%	46.7%	76.7%	90.0%	80.0%	100.0%
	LDA	75.0%	70.0%	80.0%	55.0%	43.3%	66.7%	75.0%	80.0%	70.0%
	PLS	63.3%	46.7%	80.0%	41.7%	33.3%	50.0%	80.0%	80.0%	80.0%

LDA: linear discriminating analysis; NIR: near-infrared; PLS: partial least square; SVM = support vector machine. * Raman spectra for both Day 0 and Day 7 were pulled to form a unique set of spectra.

**Table 2:** Prominent detected positive Raman peaks in each sample type.

Sample Types	Peak Wavenumber (cm^−1^)		Assignments	Compounds
	Detected *	Reported **		
Extended	328	379	Not assigned	Not assigned
	549	536	S-S stretch, symmetric skeletal vibration	Lysozyme, lactate, urea
	620	620	CC aliphatic stretch	Ascorbic acid
	708	709	C-N stretching	Albumin
	815	829	Ring breathing	Tyrosine
Pellet	331	379	Not assigned	Not assigned
	396	431	Not assigned	Not assigned
	559	536	S-S stretch, symmetric skeletal vibration	Lysozyme, lactate, urea
	633	640; 638	Ring deformation	Cytosine
	827	829	Ring breathing	n/a
	1046	1055	CN symmetric stretching	n/a
	1163	1178; 1179; 1180	CH_3_/NH_3_ rocking	Tyrosine
	1317	1317	C-H vibration	n/a
	1362	1367	Not assigned	Thymine, adenine, cytosine
Plasma	355	379	Not assigned	Not assigned
	403	488; 497	N-C-O bending	Urea
	591	536	S-S stretch, symmetric skeletal vibration	Lysozyme, lactate, urea
	901	955; 956; 957; 958; 959	PO_4_^3−^ symmetric stretching	SPH
	1158	1178; 1179; 1180	CH_3_/NH_3_ rocking	Tyrosine
	1332	1326; 1327; 1329	Ring stretching	Tyrosine
	1458	1447; 1448; 1451	CH_3_, CH_2_ bending	Tryptophan, lactate
	1559	1572; 1577	Not assigned	Guanine, adenine
	1737	1666; 1667; 1668; 1670; 1671	Polypeptide backbone	Amide I

Detected peaks * were compared to currently reported ones ** found in the literature and adapted from Das et al. [19]. n/a = not available.

**Table 3:** Dynamic of positive Raman peaks during chilled storage of boar semen.

Sample Types	Storage Duration	Peak Wavenumber (cm^−1^)		Detected in		Assignments	Compounds
		Detected *	Reported **	Good	Poor		
Extended	Day 0	588	536	P	A	S-S stretch, symmetric skeletal vibration	Lysozyme, lactate, urea
		770	768	P	P	Ring vibrations	Albumin
		1281	1267	P	P	Symmetric ring deformation/breathing	Tyrosine
		1412	1418	A	P	CH_2_ scissoring band	Lipids
		1465	1451	P	A	CH_2_, CH_3_ bend	Tryptophan, lactate
	Day 7	575	536	A	P	S-S stretch, symmetric skeletal vibration	Lysozyme, lactate, urea
		976	1001	P	A	Aromatic ring breathing	Phenylalanine
		1109	1127	A	P	CN asymmetric stretching	Tyrosine
		1342	1342	P	A	C-H bend	Tryptophan
		1550	1572	P	A	Not assigned	Adenine, guanine
		1643	1662	A	P	Amide I bend	Lysozyme
Spermatozoa	Day 0	595	536	P	A	S-S stretch, symmetric skeletal vibration	Lysozyme, lactate, urea
		770	768	P	P	Ring vibrations	Albumin
		996	1001	A	P	Aromatic ring breathing	Phenylalanine
		1104	1096	P	A	PO^4−^ stretching	PO^4−^ backbone
		1289	1268	P	P	Symmetric ring deformation	n/a
		1612	1616	A	P	C-C stretching	Tyrosine
	Day 7	779	780	P	P	ν1 symmetric stretching	Thymine, cytosine
		984	984	P	P	CH_2_ wagging	n/a
		1112	1125	P	P	CN asymmetric stretching	Tyrosine
		1297	1268	A	P	Symmetric ring deformation	n/a
		1411	1418	P	A	CH_2_ scissoring band	Lipids
Plasma	Day 0	760	762	P	P	Symmetric ring breathing	Tryptophan
		981	983	P	P	CH_2_ wagging	n/a
		1163	1178	P	A	CH_2_/NH_3_ rocking	Tyrosine
		1276	1268	A	P	Symmetric ring deformation	n/a
		1416	1418	P	A	CH_2_ scissoring	Lipids
		1580	1577	P	A	Not assigned	Adenine, guanine
	Day 7	770	768	P	P	Ring vibrations	Albumin
		981	983	P	P	CH_2_ wagging	n/a
		1312	1316	P	P	CH_2_ CH_3_ twisting or bending	Guanine
		1101	1096	P	P	PO^4−^ stretching	PO^4−^ backbone
		1419	1418	P	A	CH_2_ scissoring	Lipids
		1754	1754	P	A	C9=O ester carbonyl	Protein

Detected peaks * were compared to currently reported ones ** found in the literature and adapted from Das et al. [19]. A: absent and P: present.

**Table 4:** Characteristics of boar spermatozoa following prolonged storage at 17ºC.

Analyzed Parameters	Storage (Day)	Proportions (and AFI) of:	
		Good Samples (n = 6)	Poor Samples (n = 6)
Apoptotic cells (%)	0	2.79 ± 2.46 ^aA^ (10,480)	38.67 ± 6.51 ^aB^ (16,310)
	7	27.86 ± 25.40 ^aA^ (12,833)	14.09 ± 6.38 ^bA^ (19,185)
MD cells (%)	0	4.73 ± 4.65 ^aA^ (430)	38.41 ± 3.71 ^aB^ (398)
	7	0.48 ± 0.30 ^aA^ (1347)	60.95 ± 5.04 ^bB^ (1272)
Intracellular ROS (%)	0	5.34 ± 4.91 ^aA^ (6292)	36.21 ± 3.62 ^aB^ (6693)
	7	5.43 ± 5.42 ^aA^ (8559)	6.27 ± 2.99 ^bA^ (10,116)
MDA (µM)	0	1.58 ± 0.16 ^aA^	2.59 ± 0.50 ^aA^
	7	1.26 ± 0.00 ^aA^	1.95 ± 0.26 ^aB^
TAOC (mM)	0	2.53 ± 0.14 ^aA^	2.17 ± 0.07 ^aB^
	7	3.56 ± 0.05 ^bA^	3.17 ± 0.23 ^bA^
TAOC/MDA (×10^3^)	0	1.68 ± 0.17 ^aA^	0.98 ± 0.16 ^aB^
	7	2.82 ± 0.44 ^bA^	1.80 ± 0.29 ^aB^

Analyses were performed with freshly extended (Day 0) and chill-stored semen for seven days (Day 7) at 17 °C. Data are mean ± SEM. AFI: average fluorescent intensity (values in parenthesis) in arbitrary unit; MD: membrane damaged; MDA: malondialdehyde; ROS: reactive oxygen species; TAOC: total antioxidant capacity. ^a,b^ Different superscripts indicate significant differences during storage (Day 0 vs. Day 7) for each analyzed parameter. ^A,B^ Different superscripts indicate significant differences between Good and Poor samples for each analyzed parameter.

## Data Availability

Available data are presented in the manuscript.
